# Evaluation of satellite Leaf Area Index in California vineyards for improving water use estimation

**DOI:** 10.1007/s00271-022-00798-8

**Published:** 2022-06-09

**Authors:** Yanghui Kang, Feng Gao, Martha Anderson, William Kustas, Hector Nieto, Kyle Knipper, Yun Yang, William White, Joseph Alfieri, Alfonso Torres-Rua, Maria Mar Alsina, Arnon Karnieli

**Affiliations:** 1 Hydrology and Remote Sensing Laboratory, US Department of Agriculture, Agricultural Research Service, Beltsville, MD USA; 2 Department of Environmental Science, Policy, and Management, University of California, Berkeley, Berkeley, CA USA; 3 Complutum Tecnologías de la Información Geográfica S.L. (COMPLUTIG), Alcalá de Henares, Madrid Spain; 4 Sustainable Agricultural Water Systems, US Department of Agriculture, Agricultural Research Service, Davis, CA USA; 5 Department of Civil and Environmental Engineering, Utah State University, Logan, UT USA; 6 E&J Gallo Winery Viticulture Research, Modesto, USA; 7 Jacob Blaustein Institutes for Desert Research, Ben Gurion University of the Negev, Sede Boker Campus, Israel

## Abstract

Remote sensing estimation of evapotranspiration (ET) directly quantifies plant water consumption and provides essential information for irrigation scheduling, which is a pressing need for California vineyards as extreme droughts become more frequent. Many ET models take satellite-derived Leaf Area Index (LAI) as a major input, but how uncertainties of LAI estimations propagate to ET and the partitioning between evaporation and transpiration is poorly understood. Here we assessed six satellite-based LAI estimation approaches using Landsat and Sentinel-2 images against ground measurements from four vineyards in California and evaluated ET sensitivity to LAI in the thermal-based two-source energy balance (TSEB) model. We found that radiative transfer modeling-based approaches predicted low to medium LAI well, but they significantly underestimated high LAI in highly clumped vine canopies (RMSE ~ 0.97 to 1.27). Cubist regression models trained with ground LAI measurements from all vineyards achieved high accuracy (RMSE ~ 0.3 to 0.48), but these empirical models did not generalize well between sites. Red edge bands and the related vegetation index (VI) from the Sentinel-2 satellite contain complementary information of LAI to VIs based on near-infrared and red bands. TSEB ET was more sensitive to positive LAI biases than negative ones. Positive LAI errors of 50% resulted in up to 50% changes in ET, while negative biases of 50% in LAI caused less than 10% deviations in ET. However, even when ET changes were minimal, negative LAI errors of 50% led to up to a 40% reduction in modeled transpiration, as soil evaporation and plant transpiration responded to LAI change divergently. These findings call for careful consideration of satellite LAI uncertainties for ET modeling, especially for the partitioning of water loss between vine and soil or cover crop for effective vineyard irrigation management.

## Introduction

Water management in California faces ever-growing challenges due to limited water supplies and competing demands from agriculture, industry, and the ecosystem. Population growth and the increasingly variable precipitation patterns caused by climate change further exacerbate the water crisis. Data from the Gravity Recovery and Climate Experiment (GRACE) satellite and in situ measurements suggest that groundwater in the California Central Valley declined by 11.3 km^3^/year during 2012 and 2016 due to droughts and increased water use by irrigation (Xiao et al. 2017). The depletion is projected to continue at a higher rate in the future without mitigating efforts (Alam et al. 2019). As a result, there is an imperative need to develop efficient irrigation management strategies to reduce overdrafts and improve the resilience of the agricultural system to future climate extremes.

Irrigation is critical for vineyards to sustain plant water uptake and control berry quality (Knipper et al. 2019). To conserve water and improve water use efficiency, the frequency and amount of irrigation can be determined by monitoring evapotranspiration (ET), which quantifies the water lost from the soil through direct evaporation and plant transpiration (Ko and Piccinni 2009; Mahmoud and Gan 2019). ET can be routinely estimated using remote sensing images collected from airborne or satellite sensors (Allen et al. 2007; Anderson et al. 2012; Hoffmann et al. 2016; Knipper et al. 2020). Compared to ground-based measurements, remote sensing approaches have demonstrated capabilities to resolve between- and within-field spatial heterogeneities in plant water stress, with great potential to support the operational irrigation scheduling (Ohana-Levi et al. 2021).

Among many remote-sensing-based ET models (Anderson et al. 1997; Bastiaanssen et al. 1998; Su 2002; Allen et al. 2007), the Two-Source Energy Balance (TSEB) model is particularly suited for the unique canopy architecture of vineyards (Norman et al. 1995; Kustas and Norman 1997; Kustas et al. 2018, 2019b), where tall and highly clumped grapevine canopies are separated by wide interrows of bare soil or cover crop. TSEB uses land surface temperature (LST) and Leaf Area Index (LAI) to partition evaporative fluxes between grape canopies and interrow soil or cover crop, which could inform irrigation management to reduce water loss from soil (Nieto et al. 2019a). TSEB is regionally implemented through the Atmosphere-Land Exchange Inverse (ALEXI) model using time-differential LST measurements from geostationary satellites (Anderson et al. 1997, 2007). A disaggregation tool called DisALEXI further downscales ALEXI fluxes to sub-field levels using high-resolution images from the MODerate Resolution Imaging Spectroradiometer (MODIS) and Landsat (Anderson et al. 2004, 2011). With a widely-used image fusion technique, i.e. the Spatial and Temporal Adaptive Reflectance Fusion Model (STARFM), DisALEXI can produce 30-m ET data cubes at daily time steps (Cammalleri et al. 2013, 2014). Both TSEB and DisALEXI have been successfully applied to estimate vineyard ET across various spatial scales (Semmens et al. 2016; Anderson et al. 2019; Knipper et al. 2019, 2020; Kustas et al. 2019b; Nieto et al. 2019a, b).

LAI is a key input in TSEB for flux partitioning. LAI data can be obtained from either ground observation or satellite retrievals. Two broad categories of approaches exist to derive LAI from satellites: empirical and physical. Empirical methods establish statistical relationships between in situ LAI measurements and relevant remote sensing indicators (Baret and Guyot 1991; Broge and Leblanc 2001; Kang et al. 2016; Wang et al. 2019; Gao et al. 2021). A typical method is to build simple relationships between LAI and a Vegetation Index (VI), which is a mathematical transformation of spectral bands (Viña et al. 2011; Nguy-Robertson et al. 2012). Non-parametric regression models such as Gaussian process regression, neural networks, and support vector machines may also be used to directly exploit individual spectral bands (Verrelst et al. 2015). Physical approaches involve solving radiative transfer models for LAI based on surface reflectance measurements from satellites (Houborg and Boegh 2008; Ganguly et al. 2012). Model inversion methods include Look Up Tables (LUT), search algorithms, or machine learning models (Myneni et al. 1999; Weiss and Baret 2016). Approaches that use machine learning to invert models are sometimes called semi-physical methods. Empirical approaches are primarily applied in local study sites where LAI is measured on the ground; however, local relationships are often constrained to specific environmental settings and cannot be generalized over time or space (Kang et al. 2016). Regional to global satellite LAI products mainly use physical or semi-physical approaches, yet practical challenges remain (Baret et al. 2016; Yan et al. 2016; Kang et al. 2021).

Satellite LAI estimation is subject to uncertainties due to low signal-to-noise ratio, forward model assumptions, the ill-posed inverse retrieval, and errors in the ancillary information (Combal et al. 2003; Fernandes et al. 2014a; Fang et al. 2019; Levitan et al. 2019). Previous validation efforts show that uncertainties in satellite LAI estimations vary by data product and biome type, with RMSE values ranging from 0.19 to 2.41 (Fang et al. 2019; Brown et al. 2020, 2021). While many studies have focused on validating satellite LAI products, less is known about how errors in LAI propagate to downstream modeling applications. A few studies found that simulated carbon and water fluxes in earth system models are sensitive to LAI, and discrepancies among satellite LAI products could lead to substantial differences in estimated Gross Primary Productivity (GPP) and ET (Ryu et al. 2011; Jiang et al. 2017; Liu et al. 2018). In vineyards, the highly clumped canopy structure, diverse trellis architectures, and seasonal cover crop create additional challenges for LAI and ET estimation from satellites (Sun et al. 2017; Kustas et al. 2019b; Nieto et al. 2019a; Gao et al. 2021). Thus, it is imperative to carefully quantify LAI estimation uncertainties and understand the impact on ET modeling for sustainable water management in viticulture.

In the current study, we evaluate different empirical and physical estimation approaches for LAI based on decametric-resolution satellite images (i.e., Landsat and Sentinel-2) and assess the sensitivity of TSEB ET modeling in response to LAI uncertainty. We focus on three study sites across the California Central Valley, featuring a broad range of climate, soil conditions, trellis designs, grape varieties, and management strategies. These sites are part of the Grape Remote sensing Atmospheric Profile and Evapotranspiration eXeperiment (GRAPEX) project (Kustas et al. 2018). LAI estimation from different methods was compared to ground measurements. A sensitivity analysis assesses the impact of LAI uncertainties on TSEB ET simulations in three sites.

## Data and methods

### Study site

**Fig. 1:**
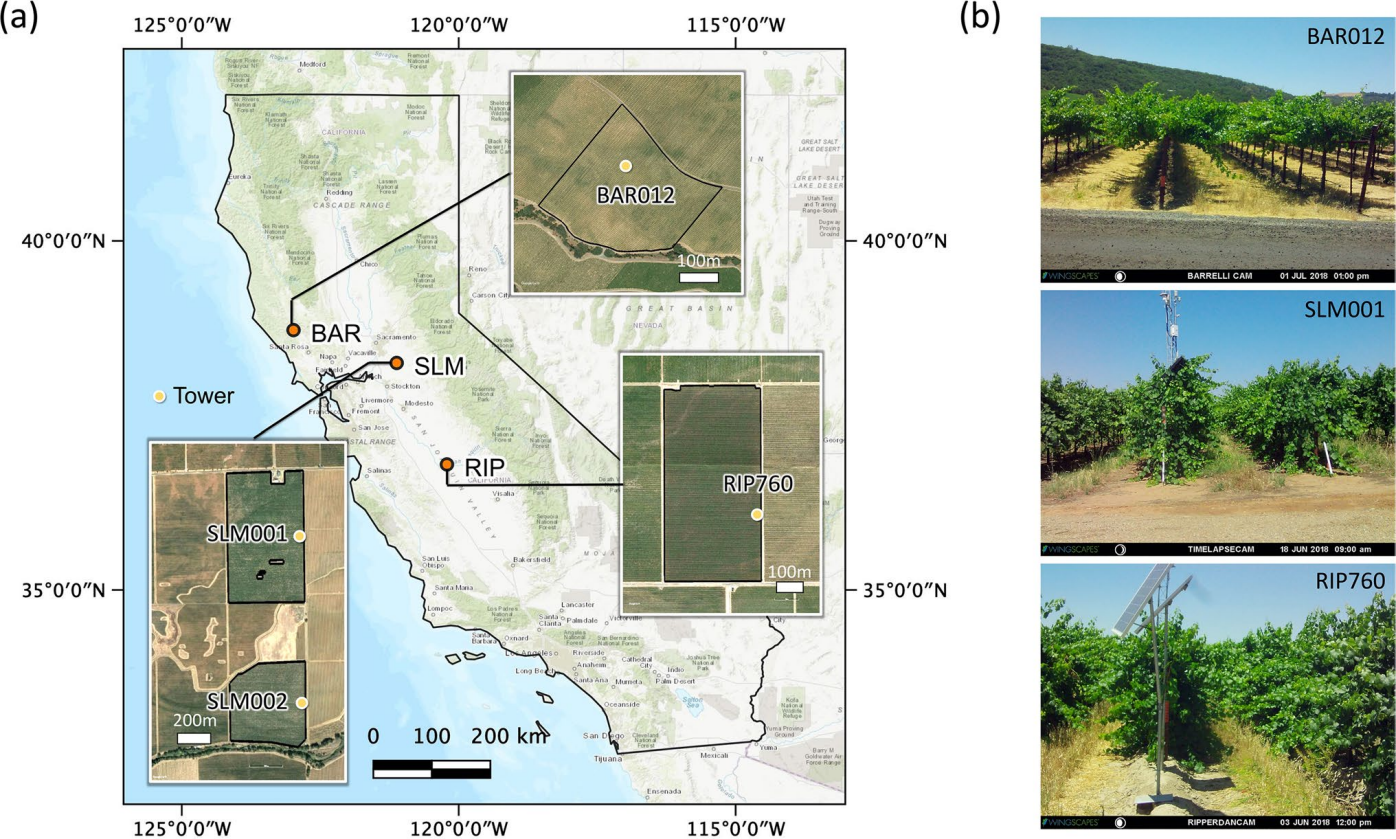
GRAPEX study site locations (**a**) and canopy snapshots (**b**). In **a**, vineyard block boundaries are outlined in the high-resolution satellite imageries (Google Earth) and solid circles indicate flux tower location. In **b**, phenocam photos for each site were selected for the peak vegetative stage in 2018

The study domain includes three GRAPEX sites in the California Central Valley: BAR, SLM, and RIP (Fig. 1, Table 1). Characteristics of these fields, including vine variety, trellis structure, and planting details, are provided in Table 1. BAR is the northernmost site close to the Pacific Ocean. In the 012 block (BAR012), vines were planted in 2010 in northeast-southwest rows (3.35 m width) with 1.83 m planting intervals. Flux tower and related measurements as part of the GRAPEX project began in 2017. In SLM, two vineyards—SLM001 (north) and SLM002 (south)—were selected as the study sites. Both fields had a 3.35 m row spacing and 1.5 m interrow spacing with an east–west row orientation. RIP block 760 (RIP760) features a double vertical trellis with a row width of 2.74 m and a planting interval of 1.83 m. The rows were planted in the east to west direction. Data collection in RIP started in 2017. All vineyards use drip irrigation. More information about these sites is detailed in Kustas et al. (2018) and Knipper et al. (2020). When evaluating LAI estimation approaches, we analyzed the results by three study sites, since the two vineyards in SLM share the same planting and trellising configurations. The TSEB sensitivity analysis was performed for each of the four vineyards using corresponding flux tower and canopy measurements.

**Table 1:** GRAPEX vineyard descriptions

Site	Location	Vineyard ID	Vine variety	Year planted	Trellising method	Row width (m)	Planting interval (m)	Year tower deployed	Measured LAI range
BAR	Sonoma, CA	BAR012	Cabernet Sauvignon	2010	Split canopy	3.35	1.83	2017	(0.8, 2.4)
SLM	Sacramento, CA	SLM001	Pinot Noir	2009	Quadrilateral	3.35	1.52	2013	(0.7, 3.9)
SLM	Sacramento, CA	SLM002	Pinot Noir	2011	Quadrilateral	3.35	1.52	2013	(1.0, 4.0)
RIP	Madera, CA	RIP760	Chardonnay	2010	Double Vertical	2.74	1.83	2017	(0.7, 4.0)

### Ground measurements

Ground measurements in GRAPEX vineyards include surface energy fluxes, downward/upward radiation, wind, temperature, precipitation, water vapor pressure, soil moisture, and routine biophysical measurements. Flux tower sensors and measurements are detailed in Kustas et al. (2018) and Knipper et al. (2020). Post-processing of the 15-min 20 Hz eddy covariance data is described in Alfieri (2019). Daytime latent heat fluxes (LE) were corrected for energy closure errors using the residual approach, i.e., adding the residual to LE, following previous studies (Semmens et al. 2016; Kustas et al. 2019b).

Biophysical measurements, including LAI, were conducted during intensive data collection periods (IOPs) at different vine and cover crop phenological stages (Kustas et al. 2018). The first IOP of each year happened shortly after bud break (flowering stage) between late April and early May. This period is generally characterized by low vine biomass and active cover crop in the interrow. The second IOP corresponds to the berry development stage (pre-veraison) in early to mid-June when vines rapidly grow while cover crops began senescence. A third IOP (mid-July to early August) occurred during the veraison stage with a fully developed vine canopy and cover crop fully senescent. A fourth IOP was conducted in late September (only during 2014). Grapes are usually harvested by late August to early September each year. IOPs were typically scheduled to coincide with Landsat overpasses.

LAI was measured using an Li-Cor LAI-2200C (in 2014–2019) or LAI-2000 (in 2013 only) instrument during IOPs (White et al. 2018). Each LAI-2200C (or LAI-2000) measurement contained one above-canopy reading and four below-canopy readings evenly placed across the interrow space. Vine LAI was determined by acquiring below canopy readings at approximately 30 cm above the ground (above cover crop). When below canopy readings were directly on the ground, the measurement included both vine and cover crop. A 45° view cap was used for all readings, i.e., 45° of the sensor field of view was exposed. From 2013 to 2016, the sensor viewing direction was parallel to the vine row. In 2016, White et al. (2018) compared multiple measurement protocols and found that a method with the sensor facing the canopy (sensor view direction perpendicular to the vine row) yielded the most consistent results with destructive measurements. Consequently, starting from 2017, all measurements were made with the sensor facing the vine row. Measurements made in 2013–2016 using the “parallel” configuration were found to underestimate LAI and were corrected by masking the two outermost rings (validated using destructive sampling) (White et al. 2018).

During IOPs, LAI was measured in grids of 25 samples (5 × 5) to the immediate west of the flux towers. The grids included five cross-row transects separated by six to seven vines, and each transect contained five measurements across five rows (White et al. 2018). LAI was also collected in other locations where sap flow and soil moisture were monitored. In these plots, LAI measurements were sampled in grids of one to three transects across five rows. The final LAI value at a location was the average of all measurements collected in the grids. Since Landsat and Sentinel-2 images have a spatial resolution at 30- or 10–20-m resolution, respectively, satellite-retrieved LAI includes both vine and crop cover. Therefore, we selected ground LAI measurements containing both vine and cover crop or vine-only measurements when the cover crop was not present (by inspecting PhenoCam photos). In total, we selected 260 ground LAI measurements with corresponding Landsat observations. Since most ground measurements in SLM were taken between 2013 and 2016, when Sentinel-2 images were not available, the number of samples was 118 for Sentinel-2 analysis.

### Satellite estimation of LAI

#### Satellite images

We derived VIs and LAI estimates from Landsat 8, Sentinel-2, and the Harmonized Landsat and Sentinel-2 (HLS) (Claverie et al. 2018) surface reflectance images that coincided with GRAPEX IOPs. We used Landsat Collection 1 Surface Reflectance images at 30 m spatial resolution. Sentinel-2 top-of-atmosphere images (L1C) were atmospherically corrected to derive surface reflectance (L2A) using the SEN2COR procedure from the Sentinel Application Platform Toolbox (SNAP) (Main-Knorn et al. 2017). The Sentinel-2 blue (B2), green (B3), red (B4), and wide-band near-infrared (B8, NIRw) are at 10 m resolution, while three red edge bands (B5, B6, B7), a narrow band NIR (B8a, NIR), and two short wave infrared (SWIR) (B11, B12) bands are at 20 m resolution. Sentinel-2 narrow-band NIR (B8a) corresponds to Landsat-8 NIR band (B5) wavelength designation. We resampled all of the 10-m bands to 20 m.

The HLS dataset combines Landsat-8 and Sentinel-2 surface reflectance data to a consistent grid (Sentinel-2 tile) and spatial resolution (30 m) through spatial co-registration, bidirectional reflectance distribution function normalization, and spectral bandpass adjustment (Claverie et al. 2018). The HLS data includes Landsat OLI-like spectral bands.

#### Vegetation indices

We evaluated the relationship between LAI and commonly used VIs: Normalized Difference Vegetation Index (NDVI), Enhanced Vegetation Index (EVI), Green Chlorophyll Index (GCI), Normalized Difference Water Index (NDWI), and Red-Edge Inflection Point (REIP) (Table 2). NDVI, EVI, GCI, and NDWI are broad-band VIs used in many previous studies to estimate LAI and other plant biophysical properties. They can be computed from both Landsat and Sentinel-2 surface reflectance images (Viña et al. 2011; Kang et al. 2016). REIP is based on red edge bands and can only be derived from Sentinel-2 images. The plant canopy absorbs red light and reflects most near-infrared (NIR) light due to the chlorophyll content in leaves and plant cell structure. The result is a sharp increase in reflectance from red to NIR. The red edge is the inflection point in the reflectance spectra between red and NIR. The shape and location of the red edge are sensitive to LAI, leaf chlorophyll content, as well as leaf hydraulic status (Horler et al. 1983; Filella and Peñuelas 1994; Darvishzadeh et al. 2009). REIP approximates the red edge with four broad red-edge bands like those from Sentinel-2. Previous studies show that REIP is a better predictor for LAI in crops than NDVI, which often saturates and becomes insensitive to high LAI values (Herrmann et al. 2011; Nguy-Robertson et al. 2014). REIP was computed from Sentinel-2 bands 4–7.

**Table 2:** Vegetation indexes evaluated in this study

Vegetation Index	Equation	References/note
Normalized Difference Vegetation index	$\mathrm{NDVI}=\frac{\mathrm{NIR}-\mathrm{Red}}{\mathrm{NIR}+\mathrm{Red}}$	Deering (1978), Wang et al. (2005)
Enhanced Vegetation Index	$\mathrm{EVI}=2.5\left(\frac{\mathrm{NIR}-\mathrm{Red}}{1+\mathrm{NIR}+6\mathrm{Red}-7.5\mathrm{Blue}}\right)$	Huete et al. (1997), Huete et al. (2002)
Green chlorophyll Index	${\mathrm{CI}}_{\mathrm{Green}}=\frac{\mathrm{NIR}}{\mathrm{Green}}-1$	Gitelson (2003, 2005)
Normalized Difference Water Index	$\mathrm{NDWI}=\frac{\mathrm{SWIR}-\mathrm{Red}}{\mathrm{SWIR}+\mathrm{Red}}$	Gao (1996), Houborg et al. (2007)
Red-edge inflection point	$\mathrm{REIP}=705+35\left[\frac{\left({\rho }_{665}+{\rho }_{783}\right)/2-{\rho }_{705}}{{\rho }_{740}-{\rho }_{705}}\right]$	Clevers et al. (2001); Herrmann et al. (2011) ${\rho }_{665}$, ${\rho }_{705}$, ${\rho }_{740}$, ${\rho }_{783}$ correspond to surface reflectance from Sentinel-2 Band 4, 5, 6, 7

* NIR* near-infrared,*SWIR* shortwave infrared

This study compared different VIs for their relationships with LAI and their prediction power of LAI in vineyards. We tested the hypothesis that the LAI-VI relationship is different across the three study sites as each has a unique combination of vine variety, canopy structure (trellis), and row configurations.

#### Physical LAI estimation approaches

Three physical/semi-physical LAI estimation methods were evaluated (methods 1–3 in Table 3): two Landsat-based approaches using reference LAI from MODIS LAI (Gao et al. 2012; Kang et al. 2021) and the Sentinel-2 Level 2 Prototype Processor (SL2P) algorithm (Weiss and Baret 2016). The Landsat-based approaches both train machine learning models using Landsat surface reflectance as predictor variables and LAI retrievals from MODIS as references. Gao et al. (2012) first proposed this scheme to generate MODIS-consistent Landsat LAI maps using the Cubist regression model and homogeneous MODIS LAI derived within a Landsat footprint over multiple years. This approach (method 1) was used by DisALEXI to generate Landsat ET maps (Yang et al. 2017; Anderson et al. 2018). Recently, Kang et al. (2021) generalized this approach over the Contiguous US (CONUS) with an advanced sample balancing strategy considering MODIS algorithm saturation and spatial, temporal, and biome representativeness across CONUS. Unlike the Gao et al. (2021) approach in which the machine learning model does not explicitly consider the biome dependence of the reflectance response to LAI, the CONUS approach trained biome-specific random forest models to represent complex vegetation conditions. This approach (method 2) is used by a Google Earth Engine (Gorelick et al. 2017) implementation of DisALEXI as part of the OpenET project (Melton et al. 2021). It is worth noting that the MODIS LAI algorithm uses eight broad plant functional types to generalize the dependence of canopy-light interactions on canopy structures. Therefore, biases arise when the actual vegetation structure differs from the model assumption, which would propagate to the two Landsat-based approaches, as they are trained on MODIS samples.

**Table 3:** LAI estimation approaches evaluated in this study

No	Description	Short name	Category	Training samples	Satellite images	References
1	Landsat-LAI based on MODIS (Local)	Landsat-MODIS (Local)	Semi-physical	MODIS (local)	Landsat	Gao et al. (2012)
2	Landsat-LAI based on MODIS (CONUS)	Landsat-MODIS (CONUS)	Semi-physical	MODIS (CONUS)	Landsat	Kang et al. (2021)
3	Sentinel-2 Level 2 Processor	SL2P	Semi-physical	Radiative transfer model simulations	Sentinel-2	Weiss and Baret (2016)
4	Landsat cubist model from ground samples	Landsat-ground	Empirical	Ground samples	Landsat	This study
5	Sentinel-2 cubist model from ground samples	Sentinel-2-ground	Empirical	Ground samples	Sentinel-2	This study
6	HLS cubist model from ground and MODIS LAI samples	HLS-ground + MODIS	Empirical	Ground samples and MODIS (local)	HLS	Gao et al. (2014); This study

The SL2P algorithm (method 3 in Table 3) estimates LAI from Sentinel-2 top-of-canopy reflectance L2A data using neural networks trained with radiative transfer simulations from the PROSPECT (Jacquemoud and Baret 1990) and SAIL model (Verhoef 1984; Fernandes et al. 2014b; Weiss and Baret 2016). Validation results show that SL2P estimation is closer to effective LAI and might underestimate LAI in clumped canopies (Djamai et al. 2019; Brown et al. 2021). While all three methods rely on machine learning models, we consider them as physical approaches since the reference LAI values were derived from radiative transfer models rather than ground measurements. Regression models serve as model inversion or search processes to connect LAI and surface reflectance. Note that MODIS LAI products were derived from Look Up Tables generated from 3-D radiative transfer models (Yan et al. 2016).

#### Empirical LAI estimation approaches

We used ground-measured LAI to build empirical models based on surface reflectance from Landsat and Sentinel-2 images (methods 4 and 5 in Table 3). Exploratory analysis suggested that LAI-VI relationships varied substantially across different vineyards and a single VI cannot provide an unbiased prediction for all sites (details in “Relationships between LAI and vegetation indices”). Therefore, we used the Cubist machine learning algorithm to establish regression models directly based on Landsat or Sentinel-2 bands. Cubist is a rule-based model with a tree structure (Quinlan 1993). Intermediate nodes include rule sets defined by input variables. Leaf nodes contain multivariate linear regression models that allow for a certain degree of extrapolation. Compared to other regression tree or random forest models, Cubist has high interpretability and performs similarly well in many remote sensing and Earth system science studies (Filgueiras et al. 2020; Kumar et al. 2021).

We built cubist models for Landsat (method 4) and Sentinel-2 (method 5) separately using ground-measured LAI from all vineyards (Table 3). A third cubist model (method 6) used the HLS dataset and combined samples from both MODIS LAI and ground measurements. The MODIS samples were screened based on their spatial homogeneity and were similar to those used in Method 1 following Gao et al. (2012). Given that MODIS LAI samples (about 50,000) cover different land cover types and outnumber ground-measured samples for vineyards (260 records), we assigned different weights to MODIS and ground samples according to the relative portion of the area they cover in the domain. For example, if vineyards covered about 3% of an HLS tile, then the total contribution of vineyard ground samples was set to 3%. The total contribution from the rest of MODIS LAI samples that include other land covers was set to 97%. The integration of in situ vineyard LAI measurements and MODIS LAI data allows capturing grapevine specific features while maintaining consistency of Landsat LAI with MODIS, which is essential for models like DisALEXI that operate across several spatial scales.

For ground-sample-only training (methods 4 and 5), we used two rules in Cubist. Exploratory analysis using five-fold cross-validation showed that increasing the number of rules beyond two may overfit and degrade model performance. Using a small number of rules also improves the model interpretability. The model with the combined ground and MODIS samples (method 6) had 30 determined by five-fold cross-validation. Additional rules were used since the LAI samples include all land cover types, not only grapevines as in methods 4 and 5. For Landsat 8 and HLS data, the input data included surface reflectance from blue (B2), green (B3), red (B4), NIR (B5), SWIR1 (B6), and SWIR2 (B7). For Sentinel-2, the input data included surface reflectance from blue (B2), green (B3), red (B4), red-edge 1 (B5), red-edge 2 (B6), red-edge 3 (B7), NIR (B8), NIR narrow (B8a), SWRI1 (B11), and SWIR2 (B12). A six-band model was also tested to be comparable to Landsat. For Landsat 8 (method 4) and HLS (method 6), we used 260 ground measurements of LAI, while for Sentinel-2 (method 5), the sample size was 118, since early measurements before 2016 did not have corresponding Sentinel-2 images. Note that since the GRAPEX IOPs were scheduled to coincide with Landsat overpass dates, most HLS data used were from Landsat 8.

#### Validation

We compared the estimation accuracy of six LAI estimation methods using ground measurements as reference (Table 3). Error metrics include root mean squared error (RMSE), mean absolute error (MAE), bias (i.e., mean difference between modeled and observed values), mean absolute percentage error (MAPE), correlation coefficient (r), and coefficient of determination (R^2^). For empirical models driven by the ground data, the accuracy was estimated using five-fold cross-validation. Since the sample size (i.e. amount of ground data) was relatively small, the cubist model could be sensitive to the way that data was split. Thus, we repeated the cross-validation procedure with different sample splitting to obtain a stable estimation of the model performance. The number of repetitions was determined statistically based on the mean and standard deviation of RMSE. An additional set of accuracy statistics was obtained using a leave-one-site-out cross-validation scheme, where one site was held out for testing and the other two were used for training. The purpose of this test was to evaluate the generalizability of empirical models.

### TSEB ET modeling and sensitivity analysis

TSEB is a land surface energy balance model that explicitly solves the convective, conductive, and radiative exchange between soil/substrate and canopy layers. TSEB has been previously applied to compute ET and partition water fluxes between soil and canopy in GRAPEX vineyards (Hoffmann et al. 2016; Kustas et al. 2019b; Nieto et al. 2019b). We used a generic version of TSEB written in Python (pyTSEB) (Nieto et al. 2021)(https://github.com/hectornieto/pyTSEB) to test the sensitivity of TSEB ET and water flux partitioning to LAI. Meteorological model inputs of TSEB include incoming and upwelling longwave radiation, air temperature, vapor pressure, and wind speed measured from eddy-covariance systems in each site. Input hemispherical LST was derived from incoming and upwelling longwave radiation following Kustas et al. (2019b) and an emissivity estimated from the assumed canopy (0.99) and soil/cover crop (0.94) emissivities weighted by fractional vegetation cover. We used hourly averages of these meteorological measurements. Major vegetation biophysical inputs included LAI and canopy height collected during IOPs. Detailed information about these measurements can be found in previous papers (White et al. 2018; Alfieri et al. 2019; Kustas et al. 2019a, b; Nieto et al. 2019a, b).

Sensitivity analysis was performed in IOP 1–4 for SLM in 2015, 2017 IOP3, 2019 IOP2 and IOP3 for BAR, and 2018 IOP 1–3 for RIP (Table 4). We used IOPs that had ground LAI measurements appropriate for remote sensing application, i.e., those that included both grapevine and cover crop, or those that were collected when cover crops were not present. For each site, we chose three to four IOPs taken during different phenological stages. For each IOP, we established a baseline TSEB output using ground measured LAI. Baseline TSEB output of daytime ET was evaluated using eddy covariance measurements, which were corrected for energy closure with the residual approach (Knipper et al. 2019). Then, sensitivity simulations were run with LAI changed by ± 5% to ± 50%, with all other inputs unchanged. The upper limit (50%) corresponded to the maximum possible satellite LAI estimation error quantified with ground measurements (“Comparison of LAI estimation methods”). Results from sensitivity simulations were compared to the baseline.

**Table 4:** List of GRAPEX IOPs used in TSEB sensitivity analysis

Vineyard ID	Year	IOP	Date	Phenological stage	Measured LAI
BAR012	2017	IOP3	08/07	Veraison	1.28
BAR012	2019	IOP2	06/25	Pea size	1.91
BAR012	2019	IOP3	07/28	Veraison	1.72
SLM001	2015	IOP1	4/23	Bloom	0.51
SLM001	2015	IOP2	6/1	Pea size	2.5
SLM001	2015	IOP3	7/9	Veraison	2.43
SLM001	2015	IOP4	8/15	Pre Harvest	2.34
SLM002	2015	IOP1	4/22	Bloom	1.04
SLM002	2015	IOP2	5/31	Pea size	2.29
SLM002	2015	IOP3	7/8	Veraison	1.77
SLM002	2015	IOP4	8/11	Pre Harvest	2.56
RIP760	2018	IOP1	6/18	Bunch Closure	3.86
RIP760	2018	IOP2	7/11	Veraison	3.78
RIP760	2018	IOP3	8/5	Pre Harvest	3.82

Since the generic TSEB model, designed for homogeneous canopies, may not fully acknowledge highly clumped vine canopies, baseline ET estimates using ground measured LAI as inputs may deviate from observed ET, so changing input LAI could either increase or decrease ET bias. Therefore, the sensitivity analysis mainly quantified the relative changes of ET and its partitioning in response to LAI changes and did not evaluate sensitivity simulations to actual ET observations. As soil evaporation approaches zero in many cases, its relative change is quantified by dividing the difference between sensitivity simulation and baseline by the average of the two. For ET and transpiration, the percentage change is the ratio between the difference of sensitivity simulation from baseline and the baseline value. Refining the TSEB model mechanism for vineyard-specific applications is a topic of active research with initial results in Kustas et al. (2019b) and Nieto et al. (2019a).

## Results

### Relationships between LAI and vegetation indices

**Fig. 2:**
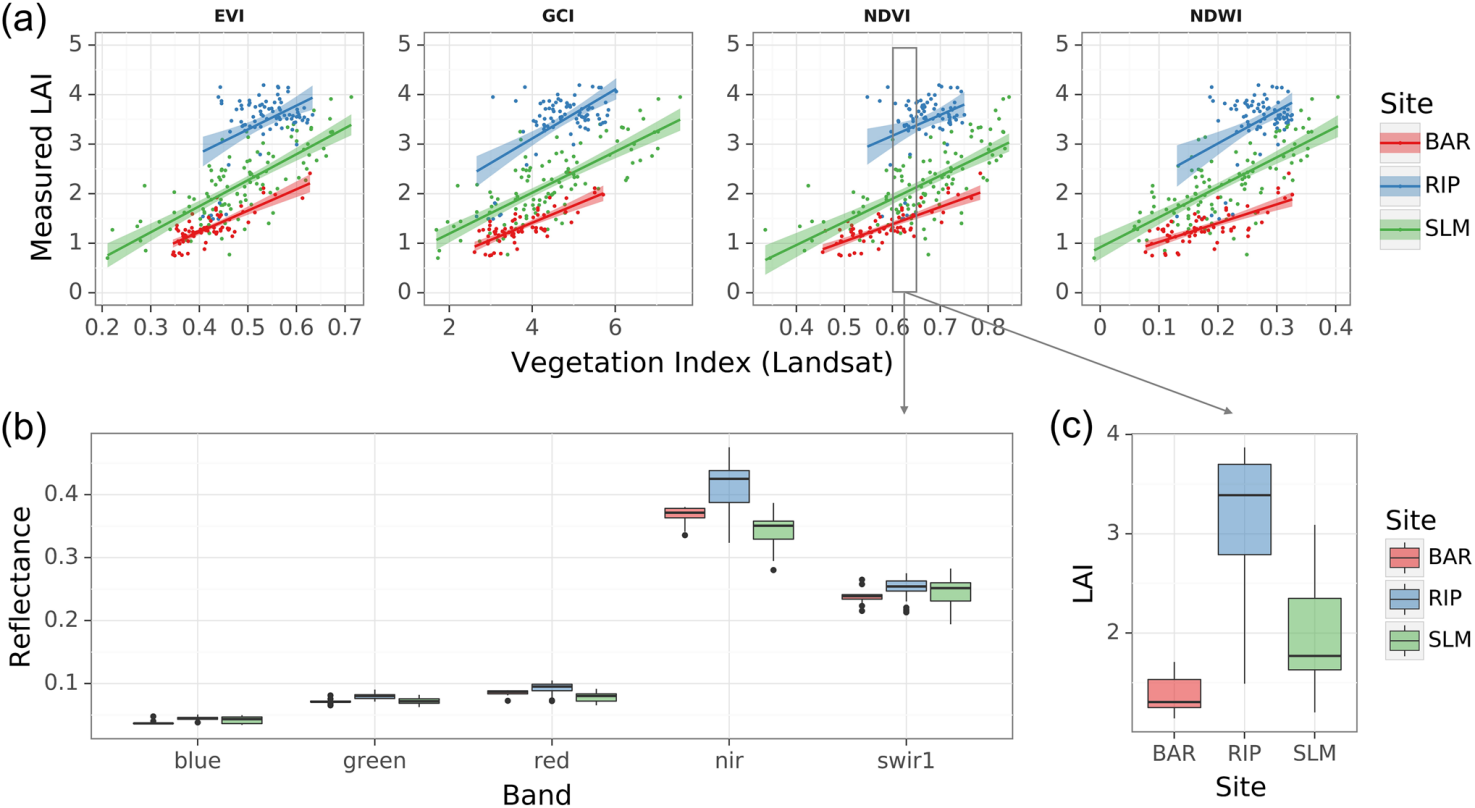
LAI-VI relationships for three GRAPEX sites based on Landsat images and ground measured LAI samples. **a** Relationships between LAI and EVI, GCI, NDVI, and NDWI for three vineyard sites. Solid lines represent fitted simple linear regression and the shaded area shows a 95% confidence interval. **b** and **c** Compares surface reflectance and LAI of samples with NDVI ranging between 0.6 and 0.65, as indicated by the grey box in (**a**), across three sites

**Fig. 3:**
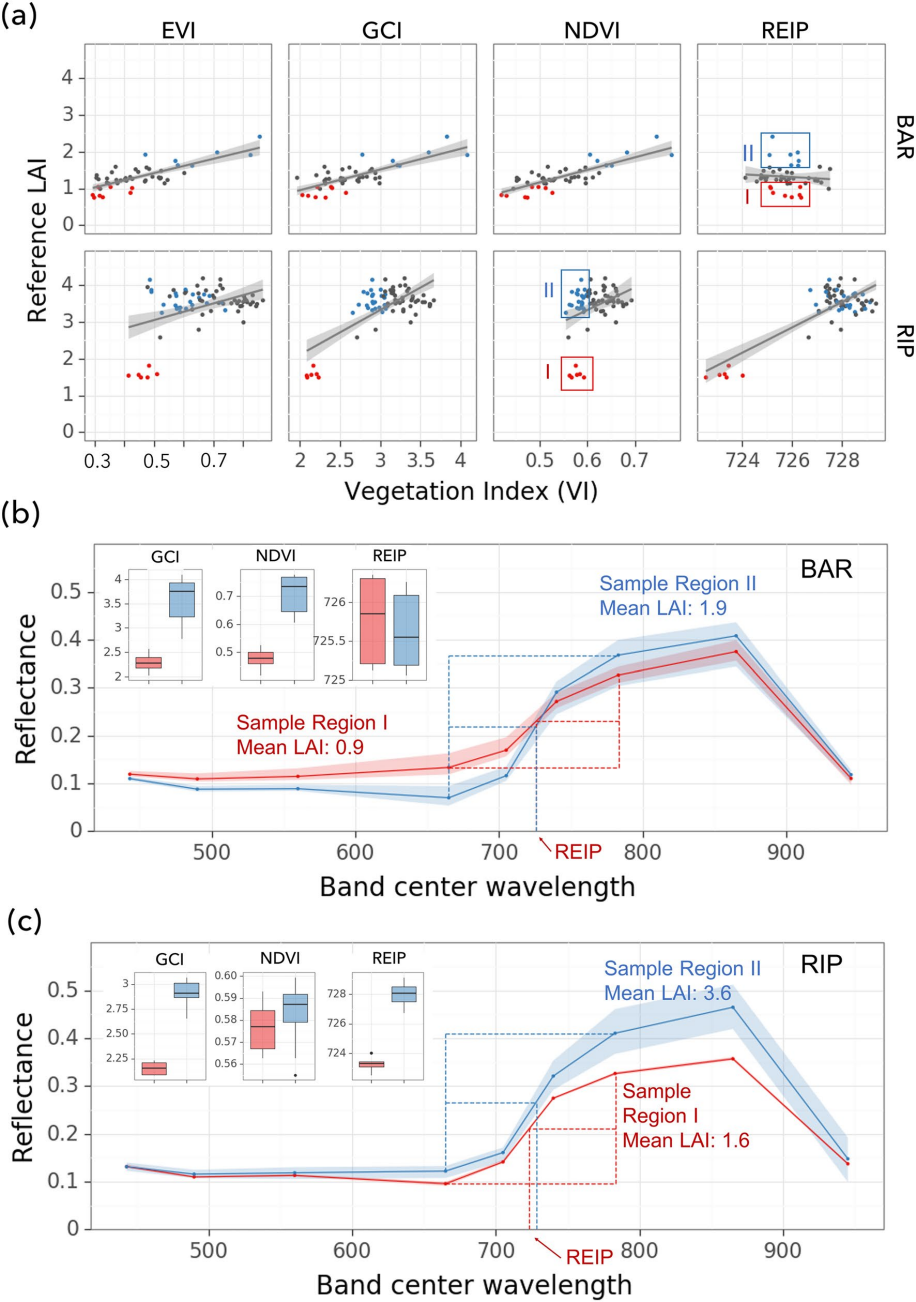
Comparison of LAI-VI relationships between REIP and other VIs (NDVI, EVI, GCI) for BAR and RIP vineyards based on Sentinel-2 images. NDWI is not presented here as its behavior is similar to NDVI and EVI. **a** LAI-VI relationships for EVI, GCI, NDVI, and REIP. For BAR (upper panel), colored points have REIP between 725 and 727 nm, but with contrasting LAI. Red points (outlined as sample region I) have LAI less than 1.2, while blue points (outlined as sample region II) have LAI greater than 1.7. For RIP (lower panel), colored points share NDVI range from 0.55 to 0.6, while red points have LAI less than 2 (sample region I) and blue points have LAI greater than 3 (sample region II). **b** and **c** illustrates the spectral profile of samples with similar VI values but contrasting LAI for BAR and RIP respectively. In **b**, red and blue lines and boxplots correspond to samples in Region I and II in the BAR (upper) panel of (**a**). In **c**, red and blue lines and boxplots correspond to samples in Region I and II outlined in the RIP (lower) panel of (**a**)

Each site presented a distinctive LAI-VI relationship for VIs extracted from both Landsat and Sentinel-2 images (Figs. 2, 3). For a given EVI, GCI, NDVI, and NDWI (Landsat) value range, LAI varied substantially across three sites with RIP being the highest and BAR being the lowest (Fig. 2a). For example, the median LAI values of samples with NDVI ranging from 0.6 to 0.65 were around 1.3, 1.7, and 3.4 for BAR, SLM, and RIP, respectively. The high LAI of RIP was likely connected to high canopy clumping because of its double-vertical trellis structure (Fig. 1). The highly contrasting responses of VI to LAI in different sites make it difficult to establish universal relationships applicable to all vineyard architectures. However, having the same VI does not ensure equality of reflectance in each band. Within samples whose NDVI values were around 0.6 to 0.65, surface reflectance in six Landsat bands had distinctive patterns for each site. For instance, RIP had a higher reflectance in red, NIR, and SWIR than the other sites (Fig. 2b). This suggests that while LAI-VI relationships do not generalize across sites, a unified model for all sites may be established by considering individual bands.

The red-edge VI—REIP from Sentinel-2 provided information on LAI complementary to that from the other VIs (Fig. 3). In RIP, the relationship between NDVI and LAI was not significant, since a wide range of LAI (1.2–4.2) samples fell into a narrow range of NDVI values (red and blue boxes in Fig. 3a). Nevertheless, REIP increased with LAI linearly. In contrast, in BAR where NDVI had a linear relationship with LAI (as did EVI and GCI), REIP had no significant relationship with LAI. The relationship for NDWI, not shown in Fig. 3, was similar to that of NDVI and EVI. These phenomena can be further understood by looking at individual bands. Figure 3a highlighted two sets of samples with contrasting LAI but similar NDVI or REIP values for RIP and BAR, respectively. In RIP, higher LAI samples (blue) had elevated reflectance when compared to lower ones in both red and NIR bands leading to only a subtle change in NDVI (Fig. 3c). However, there was a clear shift of the red edge towards a long wavelength which significantly increased REIP. In BAR, high LAI samples (red) had lower reflectance in the red band but higher reflectance in NIR, leading to a much higher NDVI compared to low LAI samples (blue) (Fig. 3b). However, the inflection point or the position of the red edge did not change. These results suggest the importance of red edge bands in detecting changes in LAI when NDVI saturates. Also, note that the GCI-LAI relationship was more significant than EVI and NDVI in RIP, highlighting the role of the green band in LAI estimation.

### Empirical model results

We used the Cubist algorithm to explore the potential of building a generalized LAI model across all vineyards using all bands rather than a single VI. A two-rule Cubist model based on six Landsat bands achieved an RMSE of 0.48 and explained 78% of the variation in ground measured LAI with no obvious bias (Table 5). Dropping the blue and SWIR2 bands significantly reduced model performance (RMSE = 0.61). Adding three VIs (NDVI, EVI, GCI) to the model slightly inflated the error (RMSE = 0.49), suggesting overfitting. When Cubist models were trained using a combination of ground and MODIS LAI samples (method 6), testing error increased for ground samples (overall RMSE: 0.53, ground RMSE: 0.59) while still within reasonable ranges. The combined model explained 69% of the variation in ground LAI, with added benefits of providing MODIS-consistent LAI estimates for all land covers, essential for DisALEXI ET modeling. Similarly, the combined model also saw degraded performance when fewer bands were involved, or when VIs were added to the feature set. In all Cubist experiments, we did not find a significant divergence in training and testing errors suggesting a low risk of overfitting (Table 6).

**Table 5:** Training and testing errors of Cubist models based on Landsat images using ground measured LAI or a combination of ground and MODIS LAI

Method	Features	Sample	Train				Test			
			RMSE	MAPE (%)	Bias	*R*^2^	RMSE	MAPE (%)	Bias	*R*^2^
Landsat-ground	Four bands (Green, Red, NIR, SWIR1)	Ground-only	0.55	21	0.03	0.71	0.61	23	0.03	0.66
Landsat-ground	All bands (Blue, Green, Red, NIR, SWIR1, SWIR2)	Ground-only	0.43	17	0.02	0.82	0.48	19	0.03	0.78
Landsat-ground	All bands + VIs (NDVI, EVI, GCI)	Ground-only	0.43	17	0.02	0.82	0.49	19	0.02	0.77
HLS-ground + MODIS	Four bands	Ground + MODIS	0.55	21	0.10	0.80	0.56	21	0.10	0.80
HLS-ground + MODIS	Four bands	Ground-only	0.49	17	0.11	0.78	0.57	19	0.14	0.70
HLS-ground + MODIS	All bands	Ground + MODIS	0.52	20	0.09	0.82	0.53	20	0.09	0.82
HLS-ground + MODIS	All bands	Ground-only	0.50	17	0.11	0.77	0.59	19	− 0.14	0.69
HLS-groud + MODIS	All bands + VIs (NDVI, EVI, GCI)	Ground + MODIS	0.52	19	0.09	0.83	0.52	20	0.09	0.82
HLS-ground + MODIS	All bands + VIs (NDVI, EVI, GCI)	Ground-only	0.48	16	0.10	0.78	0.61	19	0.13	0.66

Errors were from fivefold cross-validation with 20 repetitions. The “Sample” column refers to the subset of samples that training and testing errors were computed from. Ground measured LAI: *n* = 260. MODIS LAI samples: *n* ~ 50,000

**Table 6:** Training and testing errors of Cubist models based on a common set of ground measured LAI samples (*n* = 118)

Satellite	Features						Train				Test			
							RMSE	MAPE	Bias	R2	RMSE	MAPE	Bias	R2
Sentinel-2	All bands (Blue, Green, Red, Red-edge1, Red-edge2, Red-edge3, NIR, NIRw, SWIR1, SWIR2)						0.24	9%	-0.02	0.96	0.32	12%	− 0.03	0.92
Sentinel-2	All bands + VIs (NDVI, EVI, GCI, REIP)						0.24	8%	-0.01	0.96	0.35	13%	− 0.03	0.91
Sentinel-2	Six bands (Blue, Green, Red, NIR, SWIR1, SWIR2)						0.31	11%	-0.01	0.93	0.42	14%	− 0.02	0.87
Landsat-8	Six bands (Blue, Green, Red, NIR, SWIR1, SWIR2)						0.39	16%	-0.04	0.88	0.50	19%	− 0.04	0.82
HLS	Six bands (Blue, Green, Red, NIR, SWIR1, SWIR2)						0.35	12%	-0.01	0.91	0.49	17%	− 0.03	0.81

Errors were from fivefold cross-validation with 200 repetitions

The Cubist model based on ten bands from Sentinel-2 (method 5) achieved an RMSE of 0.32, a percentage error of 12%, and *R*^2^ of 0.92 in RIP and BAR (*n* = 118) (Table 7). Similar to the Landsat models, adding VIs (NDVI, EVI, GCI, REIP) to the Sentinel-2-based model also slightly reduced the accuracy. Further, we tested the three empirical approaches using the same ground data and same input feature set consisting of six bands that Landsat-8 and Sentinel-2 share in common. The Sentinel-2 six-band model had a higher accuracy than both Landsat-8 and HLS-MODIS + ground, with RMSE 0.42 (S2) vs. 0.49–0.50 (HLS, Landsat) and *R*^2^ 0.87 vs. 0.81–0.82 (HLS, Landsat). This is likely attributed to the higher spatial resolution (20 m vs. 30 m) and better geolocation accuracy of Sentinel-2. Meanwhile, the accuracy of the six-band Sentinel-2 model was substantially lower than the ten-band version (RMSE 0.42 vs 0.32), suggesting the value of red edge bands in measuring LAI.

**Table 7:** Leave-one-site-out cross-validation results for Cubist models based on Landsat images and ground measured LAI samples (*n* = 260)

Test site	Train				Test			
	RMSE	MAPE	Bias	R2	RMSE	MAPE	Bias	R2
BAR	0.40	13%	− 0.02	0.82	1.06	79%	0.95	0.13
RIP	0.39	18%	− 0.05	0.71	1.31	35%	− 1.20	0.21
SLM	0.33	12%	0.02	0.92	0.66	24%	− 0.39	0.53
Overall	–	–	–	–	1.01	42%	− 0.30	0.14

The feature set included six Landsat bands and the models had two rules

**Fig. 4:**
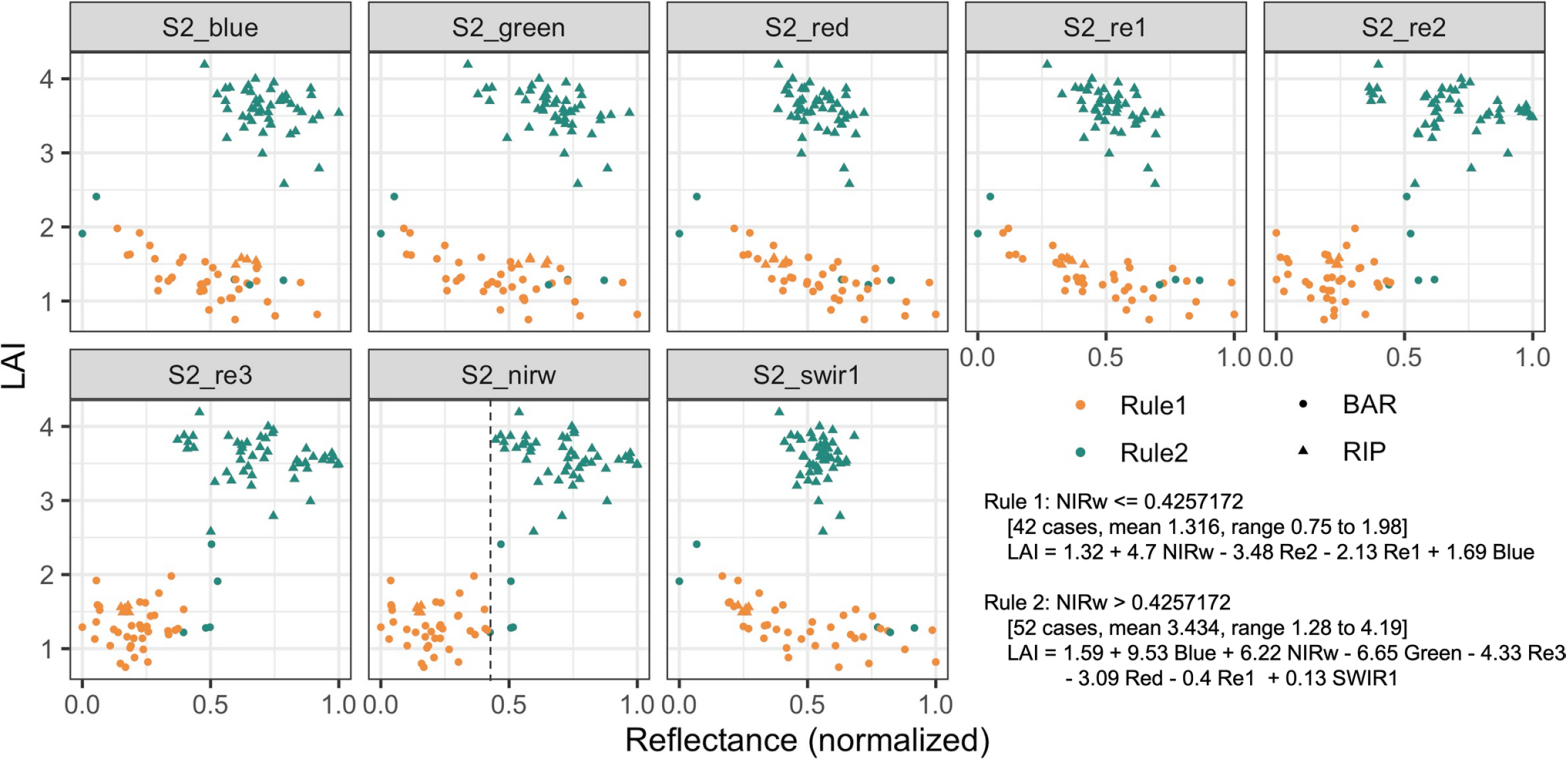
Cubist model for Sentinel-2 and scatter plots between LAI and Sentinel-2 bands. Reflectance was normalized based on max and min, so that coefficients in linear regression equations reflect the importance of input bands. Dashed line in NIRw panel indicates rule threshold. The underlying data was 80% of all samples (*n* = 118) selected as the training set. Note that while all ten bands were set as features, Cubist automatically determined features used as a rule or in the linear regression models

The two-rule Cubist models for Landsat and Sentinel-2 both used NIR (NIRw for Sentinel-2) around 0.4 as the split threshold. The exact threshold value ranged between 0.37 to 0.43 depending on the random split of training samples. Samples in the first rule had lower NIR and lower LAI, while samples in the second rule has larger NIR and higher LAI, reflecting major differences in the spectral response to LAI in sparse and dense canopies. Taking the Sentinel-2 two-rule model as an example (Fig. 4), most RIP samples were characterized by high NIRw and high LAI thus belonging to the second rule, while most BAR samples belonged to Rule 1. Both rules saw a large positive impact of NIRw on LAI (high coefficients). Red-edge 1 (Band 5) highly correlated with red, while Red-edge 2 (Band 6) and Red-edge 3 (Band 7) shared information with NIRw. There was a small negative impact of Red-edge 2 and Red-edge 3 on LAI within each rule, but overall, the impact was positive. Note that while all ten bands were pre-defined as features, Cubist automatically determines which feature(s) to use for rules or in the linear regression models. In this example, NIR (Band 8), which highly correlates with NIRw (Band 8A), and SWIR2 (Band 12), which highly correlates with SWIR1 (Band 11), were dropped by Cubist. The band selection may differ depending on the random split of the training set.

While all three empirical approaches provide robust LAI estimation with minimal bias, model performance may degrade substantially when tested in independent locations, as evidenced by the leave-one-site-out test (Table 7). RMSE of LAI reached 1.06, 1.31, and 0.66 for BAR, RIP, and SLM sites used as independent tests, respectively, mainly attributed to estimation bias. LAI in RIP and SLM were underestimated with large negative biases (-1.2 and -0.39, respectively), while RIP had a positive 0.95 bias. The direction of site-specific biases was consistent with the contrasting LAI-VI relationships and connected to canopy clumping differences among sites (Fig. 2a).

### Comparison of LAI estimation methods

**Fig. 5:**
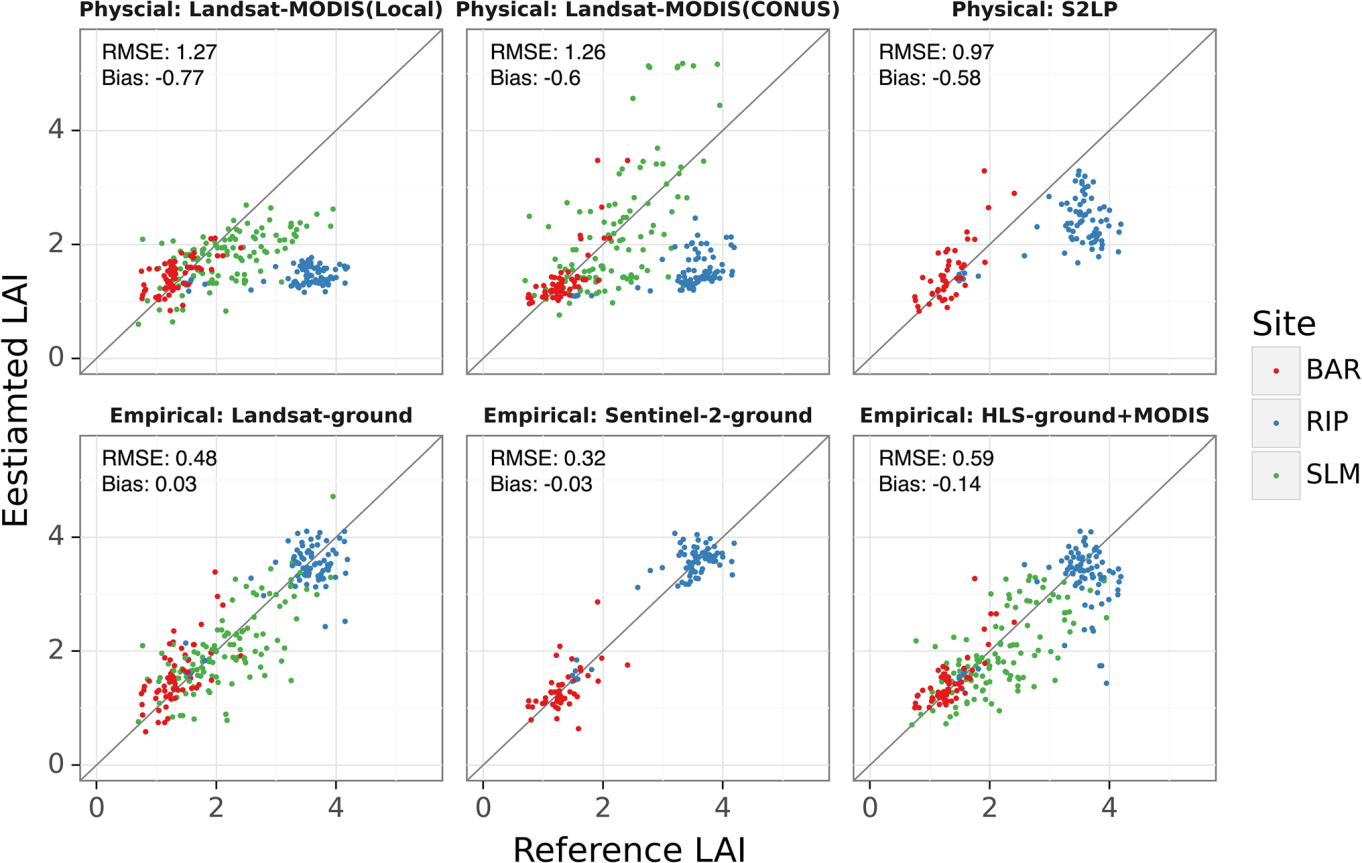
Scatter plots of ground measured LAI and predicted LAI from six estimation methods. For Landsat-based methods, the number of reference LAI samples is 260. For Sentinel-2 based methods, the number of reference LAI samples is 118. Results of empirical methods were from fivefold cross-validation

**Fig. 6:**
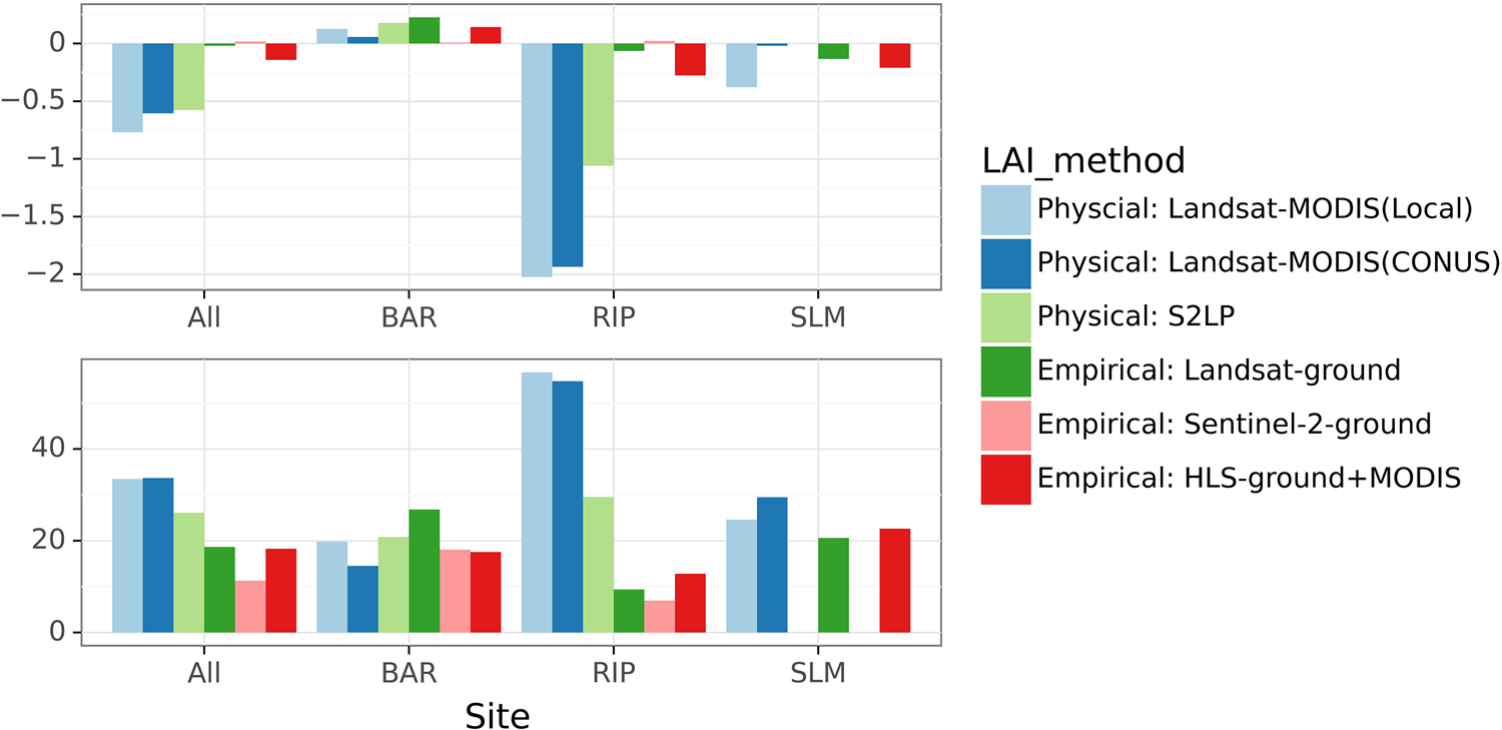
Bias and MAPE (%) of six LAI estimation methods for different sites. Errors of empirical methods were from fivefold cross validation

Three physical LAI estimation methods showed an overall RMSE between 0.97 and 1.27, and the Sentinel S2LP algorithm achieved the highest accuracy, while two Landsat-based approaches performed similarly (Table 8). All three underestimated LAI in RIP (Figs. 5, 6). The local Landsat-MODIS algorithm also underestimated medium LAI (values between 2 and 4) in SLM. Although the overall accuracies of both Landsat-based methods were similar, the CONUS approach showed a smaller bias for high LAI (e.g. In SLM and RIP) attributed to the balanced sampling strategy considering both unsaturated and saturated MODIS LAI (Kang et al. 2021). But the CONUS approach was also associated with a lower precision as seen in Fig. 5. The local scheme had a higher accuracy for LAI below 2.

**Table 8:** Comparison of six LAI estimation methods

Method no	Category	LAI_method	Count	RMSE	Bias	MAE	MAPE (%)	*r*	*R*^2^
1	Physical	Landsat-MODIS(Local)	260	1.27	− 0.77	0.93	33.47	0.19	− 0.57
2	Physical	Landsat-MODIS(CONUS)	260	1.26	− 0.60	0.93	33.65	0.30	− 0.54
3	Physical	S2LP	118	0.97	− 0.58	0.76	26.11	0.77	0.30
4	Empirical	Landsat-ground	260	0.48	− 0.02	0.36	18.64	0.88	0.77
4	Empirical	Landsat-ground (Leave-one-site-out)	260	1.01	− 0.30	0.86	41.74	0.38	0.14
5	Empirical	Sentinel-2-ground	118	0.31	0.02	0.24	11.30	0.96	0.93
6	Empirical	HLS-ground + MODIS	260	0.57	− 0.14	0.41	18.24	0.84	0.68

Errors of empirical methods were from fivefold cross-validation unless indicated otherwise

The Sentinel S2LP algorithm had the highest accuracy among the three when the comparison was made using a common set of samples, yielding RMSE = 0.97 (S2LP) vs. 1.6 (Landsat) (Table 9). This difference was mainly driven by samples from RIP, where S2LP-estimated LAI was significantly higher than Landsat LAI. The negative bias of S2LP in RIP was only half of that from Landsat estimations, but it was still considerably large ~ − 1. The underestimation of high LAI in physical models was related to several well-known reasons, with NIR reflectance saturation and model assumptions/generalizations being the most important (Myneni et al. 1999; Fang et al. 2019; Brown et al. 2021; Kang et al. 2021). Plant reflectivity of NIR light saturates and becomes insensitive to LAI as the vegetation canopy grows denser (LAI > 2–3). The intensity of this phenomenon varies according to canopy biophysical, biochemical, and structural differences. Radiative transfer models underlying global/regional LAI products often need to make simplified assumptions regarding canopy and soil properties to support generalization over large areas. As a result, estimation bias in LAI arises when canopy conditions diverge from model assumptions (i.e. a horizontally continuous canopy layer with randomly placed leaves). For grapevines, the diverse trellis designs—varying canopy geometry and clumping—creates additional challenges for remote estimation of LAI (Figs. 1, 2).

**Table 9:** Comparison of three physical LAI estimation methods with a common set of ground observations (*n* = 118)

Site	LAI_method	Count	RMSE	Bias	MAE	MAPE(%)	*r*	*R*^2^
ALL	Landsat-MODIS(Local)	118	1.64	− 1.18	1.31	42.08	0.12	− 1.04
ALL	Landsat-MODIS(CONUS)	118	1.60	− 1.16	1.28	40.58	0.29	− 0.93
ALL	S2LP	118	0.97	− 0.58	0.76	26.11	0.77	0.30
BAR	Landsat-MODIS(Local)	46	0.29	0.11	0.23	19.57	0.61	0.19
BAR	Landsat-MODIS(CONUS)	46	0.37	0.09	0.24	17.11	0.77	− 0.26
BAR	S2LP	46	0.38	0.18	0.28	20.79	0.78	− 0.32
RIP	Landsat-MODIS(Local)	72	2.09	− 2.00	2.00	56.47	0.26	− 10.40
RIP	Landsat-MODIS(CONUS)	72	2.03	− 1.95	1.95	55.58	0.45	− 9.70
RIP	S2LP	72	1.20	− 1.06	1.06	29.51	0.50	− 2.75

Estimation bias that arose from model assumptions could sometimes be mitigated by incorporating ground-measured LAI. The three empirically trained Cubist models achieved higher accuracy than physical approaches with no appreciable bias (Table 8, Figs. 5, 6). The RMSE of empirical methods ranges between 0.31 and 0.57 with MAPE below 20%, while the MAPE of physical methods could be as high as 50% (for RIP). However, empirical models may not generalize well to unknown conditions. The leave-one-site-out testing accuracy for the empirical models of Landsat was comparable to that of physical approaches, with RMSE around 1 and MAPE around 42% (Table 8).

### Impacts of LAI uncertainties on ET modeling

**Fig. 7:**
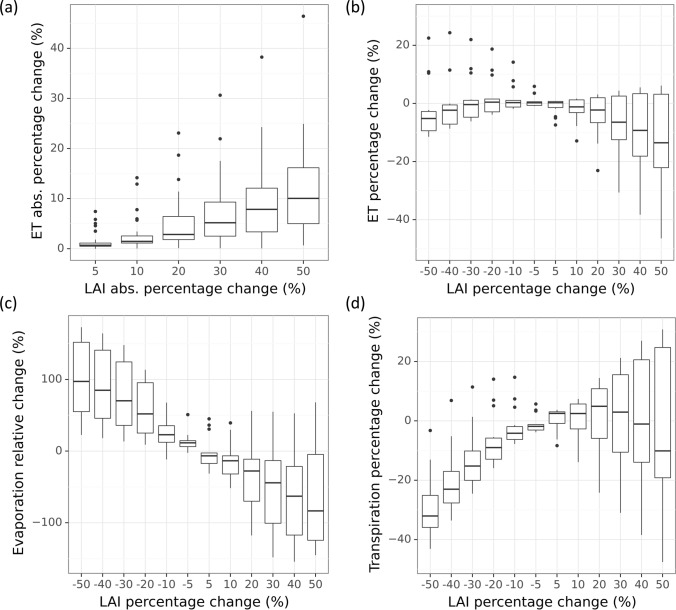
Sensitivity of ET and its partitioning to LAI estimation errors from 5 to 50%. **a** Absolute percentage change in ET vs. absolution percentage change in LAI. **b** Percent changes in ET vs. percentage changes in LAI. **c** Relative change in evaporation to LAI. Since evaporation was close to zero in many cases, relative change was quantified as dividing the difference between sensitivity simulation and baseline by their average values. **d** Percentage change in transpiration to LAI. In a boxplot, the middle bar presents the median, the box covers 25% and 75% percentiles, and the difference between the two is called the inter-quartile range (IQR). The whiskers extend from the box to 1.5 * IQR, and data beyond the whiskers are outliers and drawn individually

We analyzed the impact of LAI estimation uncertainty on TSEB ET simulation by adding ± 5 to 50% error to ground measured LAI for each IOP. Baseline TSEB daytime ET simulations forced by ground-measured LAI were consistent with eddy covariance measured ET. The RMSE was 0.55 mm/day, the mean absolute percentage error was 14%, and r^2^ was 0.78 over 14 IOPs (Table 4). Sensitivity simulations with varied errors in LAI were compared to the baseline. Absolute percent changes of TSEB ET generally increased with LAI error but with a lower magnitude (Fig. 7a). A 20% change in LAI led to less than 15% change in ET, with a median value below 5%. A 50% change in LAI contributed to less than 25% change in ET with a median of 10%. The impact of LAI on TSEB ET was asymmetric in magnitude (Fig. 7b). A positive error in LAI (i.e. LAI overestimation) was far more influential on ET than a negative one (i.e. when LAI is underestimated). A small underestimation (− 5 to − 30% in relative bias) in LAI could either increase or reduce ET, but both positive and large negative biases in LAI were more likely to reduce ET. A 50% underestimation in LAI may only lead to a small negative bias in ET up to -10%, but a 50% overestimation in LAI could reduce ET by up to 50% (the median is around 18%).

**Fig. 8:**
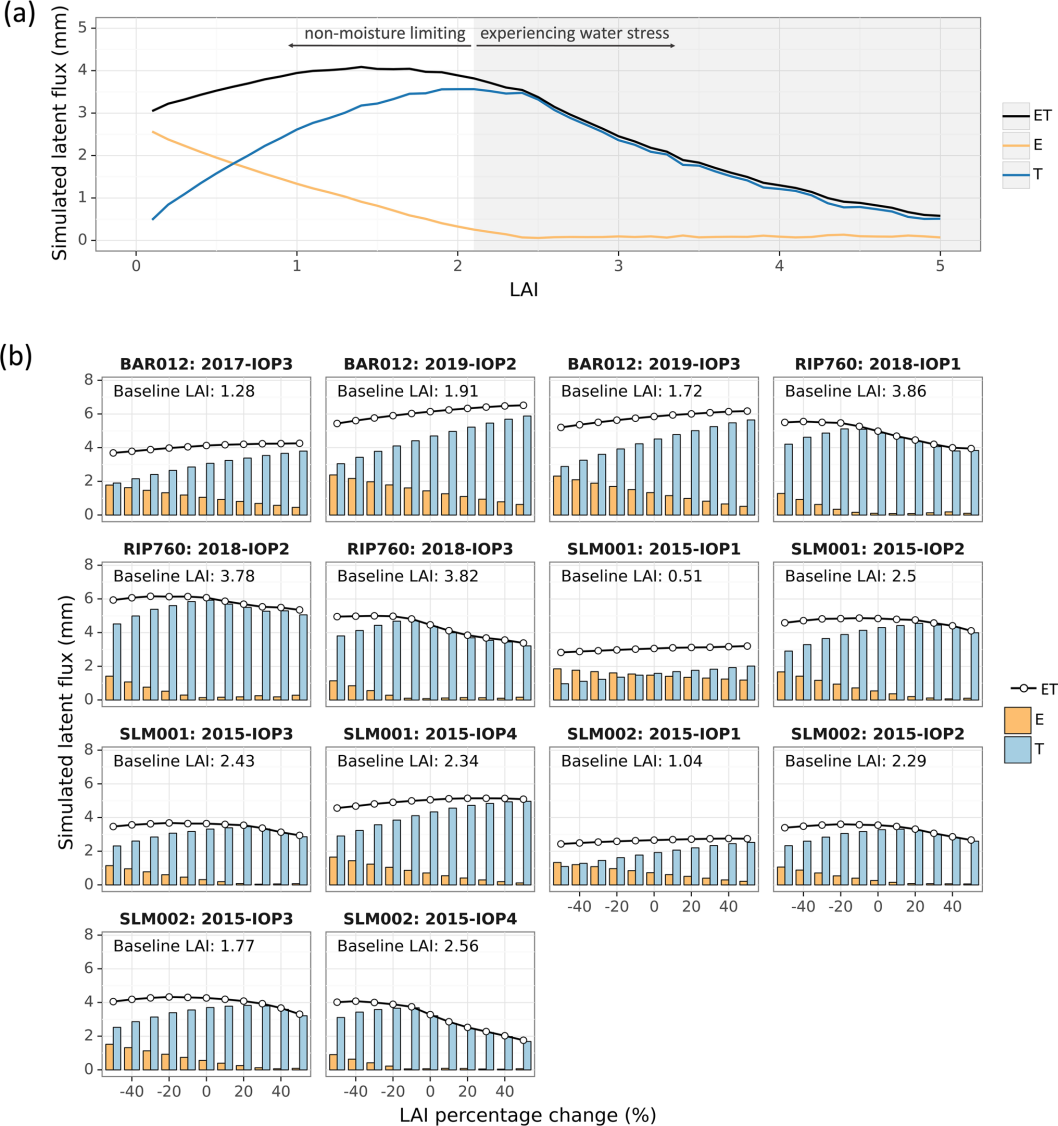
Effects of LAI estimation error on TSEB soil evaporation (E) and plant transpiration (T) partitioning. **a** Illustration of TSEB E, T, and ET responses to LAI, based on SLM002 2015-IOP2. **b** TSEB E, T, and ET response to percentage changes in LAI from baseline in selected IOPs of four vineyards. Baseline LAI refers to the field measurement

The impact of LAI on ET partitioning was further evaluated (Fig. 7c, d). While ET showed minimal responses to negative changes in LAI, evaporation (E) and transpiration (T) changes were substantial. A 50% underestimation in LAI led to a reduction in T by up to 40%. Soil evaporation generally decreases when LAI increases. However, the effect of LAI on transpiration and total ET is more complex and depends on canopy growth stages (Fig. 8a). When the vine canopy is relatively sparse (baseline LAI < 1.5), transpiration increases with LAI, outpacing the decrease in evaporation and leading to a slow increase in ET. Sensitivity simulations of three IOPs in BAR and IOP1 for two SLM vineyards belong to this case (Fig. 8b). As the vine canopy grows denser, the increase in transpiration tapers off and eventually is outpaced by the reduction of soil evaporation leading to an overall ET decrease. As LAI further increases, it starts reducing transpiration leading ET to decline at a higher rate, while soil evaporation is driven down to zero (Fig. 8a). This scenario is evident in three IOPs of RIP, and IOP2-4 for SLM (Fig. 8b). The nonmonotonic response of ET to LAI gives rise to model equifinality, where different LAI values could result in the same ET due to different partitioning between E and T.

From a modeling perspective, TSEB starts with an initial guess that the canopy is transpiring at a potential rate (non-moisture limiting) estimated using the Priestley-Taylor relationship applied to the canopy net radiation divergence (Kustas et al. 2019a). TSEB then solves for soil evaporation based on energy balance. If this results in negative soil evaporation, which is unlikely midday when Landsat TIR imagery is collected, the TSEB considers the canopy to be under stress and iteratively reduces transpiration until evaporation estimation is realistic (non-negative). Elevated LAI can serve to emulate this signal of stress by overestimating potential transpiration and thereby producing negative estimates of soil evaporation. As such, we found that transpiration degraded almost linearly with LAI when LAI was positively biased from a high baseline (Fig. 8b, three IOPs for RIP, IOP2-4 for SLM).

**Fig. 9:**
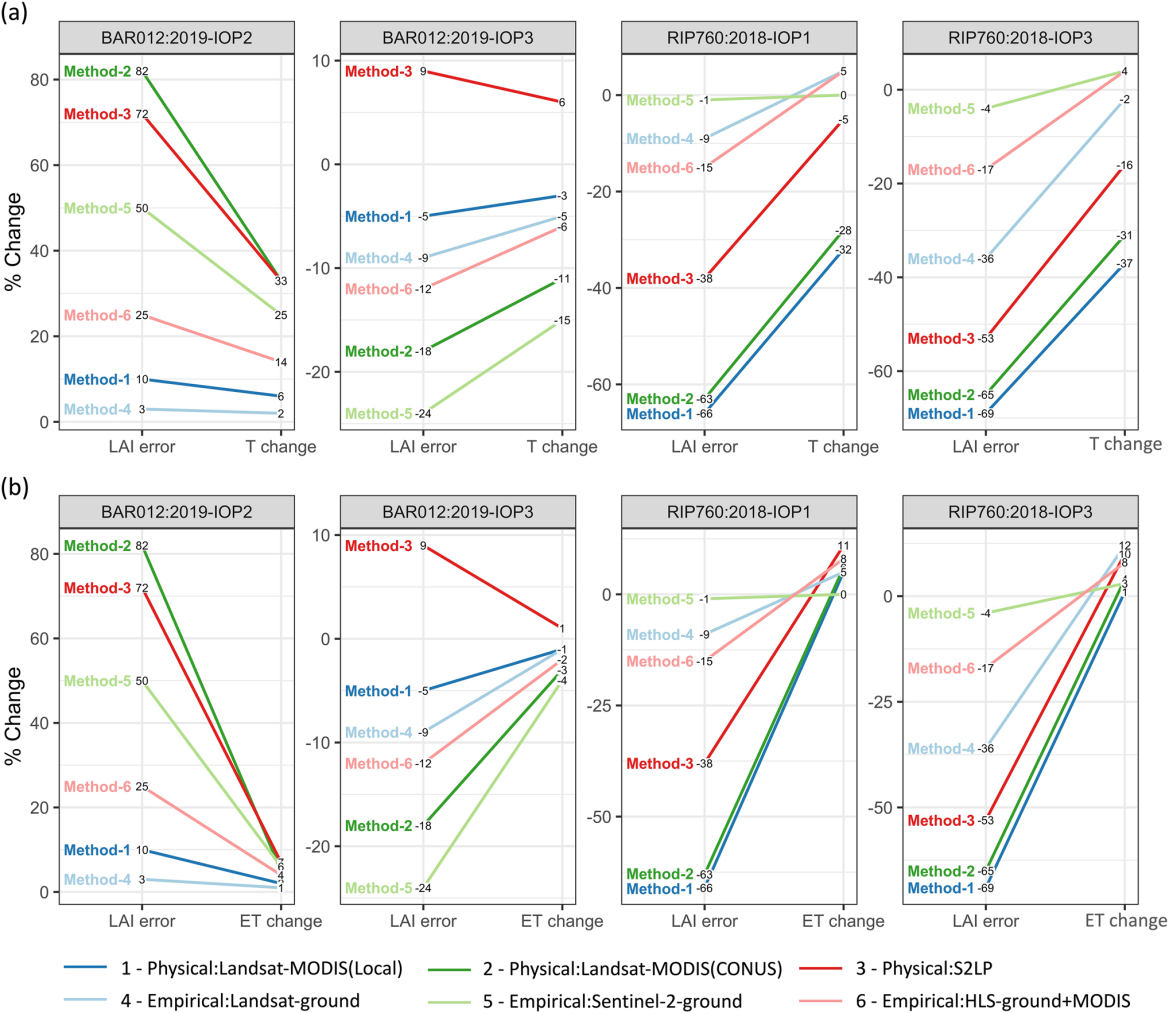
Percentage changes of transpiration (**a**) and ET (**b**) due to LAI percentage errors from six remote sensing LAI estimation methods

The exact turning points when T and ET start decreasing with LAI depend on the land surface temperature, plant, atmospheric, and soil conditions (Kustas and Norman 1997). Consequently, the sign and magnitude of estimated T and ET changes induced by LAI are functions of a series of variables besides LAI. Figure 9 shows the percentage changes in T and ET connected to LAI errors from six remote sensing LAI methods. In general, due to divergent responses of E and T to LAI, ET was not as sensitive to LAI as T. In the two IOPs of BAR012, since the baseline LAI was low (< 2), T and ET had not reached their turning point with 50% LAI errors. Their responses to LAI were mainly monotonic. In RIP IOP1 and IOP3 where the baseline LAI was high (~ 3.8), the response of T and ET to LAI errors were not monotonic (Fig. 8b). For example, in RIP IOP3, methods 1 and 2 underestimated LAI by 50–70%, but ET estimates were close to observations (< 5%). In contrast, methods 3 and 4 had lower LAI errors (30–50%) but higher ET biases (10–12%).

## Discussions

Canopy spectral response to LAI differed significantly across vineyards characterized by different planting configurations and trellis structures, consistent with previous investigations (Nguy-Robertson et al. 2014; Kang et al. 2016). While LAI-VI relationships were not universal across the three vineyards, a simple rule-based regression model reproduced LAI with no obvious bias and captured 78% of variability for all vineyards by exploiting all bands. This suggests that ratio-based VIs like EVI and NDVI often ignore differences in the absolute reflectance of individual bands like NIR and Green, which encode information on canopy structure and LAI (Badgley et al. 2017). Additionally, we found that the red-edge-based VI (REIP) from Sentinel-2 images provided complementary information to NDVI in LAI estimation (Herrmann et al. 2011). These observations imply the value of exploiting individual bands to estimate LAI across heterogeneous canopy structures rather than using a single or a few VIs that are highly inter-correlated (Gutman et al. 2021). Many remote sensing ET models estimate LAI or vegetation fractional cover through a single relationship with NDVI (Allen et al. 2011), ignoring the effect of canopy structure and other properties on canopy radiative response. Future research may benefit from deriving simple theoretical formulas directly based on reflectance across the entire spectrum including the red-edge bands.

Three physical/semi-physical approaches based on Landsat or Sentinel-2 reflectances significantly underestimated LAI for dense and highly clumped canopies (LAI > 2). Inverting radiative transfer models to retrieve LAI is an ill-posed problem (Combal et al. 2003). Radiative transfer models often use simplified assumptions to generalize across global ecosystems, thus large bias and uncertainty are unavoidable in heterogeneous landscapes, like the highly clumped canopies in vineyards and other orchards. For example, S2LP uses the turbid-medium PROSAIL model where the vegetation canopy is modeled as a horizontally homogenous green turbid medium without foliage clumping. As a result, Sentinel-2 LAI has been found to represent effective LAI rather than the true LAI (Djamai et al. 2019; Brown et al. 2021). Likewise, we compared S2LP LAI to effective LAI measured in RIP and the accuracy was significantly better with no obvious bias than when compared to true LAI (RMSE = 0.56; bias = − 0.04). The MODIS LAI algorithm used 3-D radiative transfer models (2-D for some biomes) to represent eight biomes, requiring land cover information to regulate the models. However, these are broad categories of plant functional types. Thus, specialty crops such as orchards are not well-represented. Major improvement in remote sensing estimation of LAI in the future will likely require additional canopy information to regularize the ill-posed problem, such as multi-angular (Liu et al. 2014), hyperspectral (Cawse-Nicholson et al. 2021), and LiDAR (Potapov et al. 2021) observations.

Uncertainties in LAI propagate proportionally to ET estimation in TSEB model in general, but the underlying mechanism differs across growth stages and stress conditions. When vine canopies were in early growing stages, up to 50% error in LAI (~ 0.5) did not cause a significant change in ET, as effects on E and T canceled out (Fig. 8, column 1). But individual uncertainty of E and T was around 0.5–0.8 mm with an LAI error of 0.5, which is typical of satellite retrievals. Such model equifinality (Beven 2006) may not sufficiently inform precise irrigation scheduling which requires accurate estimation of water loss both from vines and the cover crop/bare soil. For vines in late vegetative or reproductive stages, a positive bias in LAI may lead to a significant underestimation of T, as TSEB down-regulates latent heat loss to avoid energy imbalance in response to water stress. But since E responds positively to an increased LAI, the uncertainty in LAI may not propagate to ET. Taking RIP—IOP2 as an example, a negative 50% error in LAI (equivalent to the estimation from Landsat-MODIS physical approaches) reduced T by 1.4 mm, but an equivalent yet positive change was found in E. Therefore, ET change was negligible (Fig. 8). Although remote sensing estimation of LAI is more likely to underestimate rather than overestimate in dense vegetation due to the saturation issue, thus resulting in a relatively small error in ET, caution is required when water stress is assessed for vine and cover crop separately to inform irrigation scheduling (Kustas et al. 2019b; Bellvert et al. 2020).

## Conclusions

This study quantified the uncertainties of several satellite-based LAI estimation approaches and analyzed the impact of LAI errors on TSEB ET modeling for four California vineyards across climate gradients. Physical approaches for Landsat and Sentinel-2 predicted low to medium LAI reasonably well but underestimated medium to high LAI by 1 to 2 units. Sentinel-2 S2LP algorithm outperformed two Landsat-based approaches in a highly clumped vineyard. Although LAI-VI relationships differed substantially across vineyards, a unified rule-based regression model based on ground LAI measurements and surface reflectance from multiple bands achieved high estimation accuracy (RMSE ~ 0.5) with no significant bias in all vineyards. Moreover, the superior performance of Sentinel-2 approaches, both physical and empirical, highlighted the unique value of red-edge bands for LAI modeling.

ET uncertainty was generally proportional to LAI errors, but positive LAI biases more significantly affected ET than negatives ones. In moderate or dense vine canopies, positive bias in LAI led to severe water stress in TSEB simulations, which down-regulated transpiration resulting in underestimation of ET. In other cases, the overall impact of LAI uncertainties on ET may be small, but errors in E and T partitioning could be sizable due to the divergent responses of plant transpiration and soil evaporation to LAI. Precise irrigation management in vineyards requires accurate partitioning of water fluxes to improve water use efficiency and reduce water loss from soil or cover crop. Applications of remote sensing-based-ET models should be mindful of uncertainties in remote sensing LAI estimation and impacts on ET modeling, especially on E and T partitioning. Vineyard irrigation scheduling can benefit from empirical or locally calibrated physical LAI approaches based on ground measurements.
